# Experimental and Numerical Simulation Study of Oxygen Transport in Proton Exchange Membrane Fuel Cells at Intermediate Temperatures (80 °C–120 °C)

**DOI:** 10.3390/membranes14040072

**Published:** 2024-03-22

**Authors:** Jian Zhang, Yunfei Zhang, Zhengrui Xiao, Jinting Tan, Haining Zhang, Jun Yu

**Affiliations:** 1 State Key Laboratory of Advanced Technology for Materials Synthesis and Processing, Wuhan University of Technology, Wuhan 430070, China; 2 Key Laboratory of Fuel Cell Technology of Hubei Province, Wuhan University of Technology, Wuhan 430070, China

**Keywords:** PEMFC, intermediate temperature, oxygen transport, local transport resistance, numerical simulation

## Abstract

Investigating the oxygen transport law within the Membrane Electrode Assembly at intermediate temperatures (80–120 °C) is crucial for enhancing fuel cell efficiency. This study analyzed the resistance to oxygen transport within the Membrane Electrode Assembly at intermediate temperatures using limiting current density and electrochemical impedance spectroscopy. The study findings reveal that, as temperature progressively increases, the Ostwald ripening effect leads to a 34% rise in the local oxygen transport resistance (R_local_) in relation to the pressure-independent resistance (R_np_) within the cathode catalytic layer. Concurrently, the total transport resistance (R_total_) decreases from 27.8% to 37.5% due to an increase in the gas diffusion coefficient and molecular reactivity; additionally, there is a decrease in the amount of liquid water inside the membrane electrode. A three-dimensional multiphysics field steady-state model was also established. The model demonstrates that the decrease in oxygen partial pressure can be mitigated effectively by increasing the back pressure at intermediate temperatures to ensure the cell’s performance.

## 1. Introduction

Proton Exchange Membrane Fuel Cells (PEMFCs) have attracted worldwide attention for their low emissions, high energy efficiency, fast start-up, and environmental friendliness. Recently, Japan’s “FCV HDV Fuel Cell Technology Development Roadmap” announced the 2040 fuel cell development plan for heavy-duty vehicles, which will gradually raise the operating temperature to intermediate temperature [1,2]. Running fuel cells at intermediate temperatures can improve Oxygen reduction reaction (ORR) efficiency [3], increase the resistance of Pt-based electrocatalysts to CO poisoning, and simplify the water management of the cell and thermal management, among many other benefits [4,5].

To enhance the power density of the PEMFC at intermediate temperatures, it is necessary to maintain a high voltage at high current densities [6]. A significant resistance to oxygen transfer can impede oxygen transfer, resulting in low oxygen concentration at the cathode and voltage loss; therefore, improving oxygen transfer in PEMFC is crucial for increasing power density [7]. Baker [8] calculated the total resistance to oxygen transport by using limiting currents. They varied the thickness of the gas diffusion barrier (GDB) and microporous layer (MPL), dividing the resistance into pressure-dependent and pressure-independent resistance. Their findings suggest that half of the resistance to oxygen transport originates from the GDB [9]. Nonoyama [10] analyzed the gas diffusion modes in detail, identifying three in particular: molecular diffusion, Knudsen diffusion, and permeation of ionomer films. They interpreted the pressure-independent resistance of the catalytic layer (CL) as the bulk-phase resistance and the local oxygen transport resistance. Oh [11] et al. showed that oxygen transport in the GDB is dominated by molecular diffusion, while the MPL is controlled by a combination of molecular diffusion and Knudsen diffusion. Transport in the CL consists of molecular diffusion, Knudsen diffusion, and oxygen permeation at the ionomer. The study quantifies the detailed oxygen transport impedance of each component in the PEMFC.

When the temperature is increased to an intermediate level, the mass transfer of gas, substance transport, and electrochemical reactions inside the cell are altered to varying degrees. Xu [12] investigated the performance of fuel cells at different levels of humidity and found that, at low humidity, the ionomers of the CL can cause oxygen diffusion problems due to their reduced oxygen permeability. The resulting resistance to oxygen transport caused a voltage loss of 105 mV at 120 °C and 20% RH for a single cell operating at 600 mA cm^2^. Akitomo [13] investigated the impact of high pressure on the performance of the cell under operating conditions of 60–100 °C and 80% RH. The results showed that pressurization could increase the partial pressure of oxygen, thereby improving the performance of the intermediate-temperature cell. Additionally, the state of water in the MEA was analyzed in the pressurized state using the X-ray technique. The data revealed that the MPL in the MEA had a greater effect on oxygen transfer compared to the GDB. Fernihough et al. [14] evaluated the performance of PEMFC equipped with Nafion 211 membranes(Purchased from Wuhan WUT HyPower Technology Co., Ltd., Wuhan, China) at temperatures ranging from 80 °C to 120 °C. The highest performance of 1400 mA cm^−2^ was obtained at 100 °C, 100% RH, and 0.6 V. The experimental results showed that the empirical equation of water content versus membrane material activity is still applicable at intermediate temperatures. Their model is in good agreement with the polarization curves at intermediate temperatures. The experimental results in the ohmic polarization region are also in good agreement.

Observing reactions inside cells from a macroscopic point of view is challenging due to size limitations and other factors. Simulations can be used to better analyze the internal conditions of the cell. He [15] modeled a high-temperature fuel cell by combining the equations of mechanics and Butler−Volmer to simulate the trend of oxygen concentration in different diffusion layers. Xia et al. [16] developed a three-dimensional non-isothermal model to investigate the effect of the ratio of the flow channel (CH) to the rib width on the diffusion of reactive gases in porous electrodes at an operating temperature of 180 °C. The results showed that increasing the ratio helps to improve the gas transport in the electrode.

Currently, there is an increasing number of studies related to the performance of MEA for PEMFC at intermediate temperatures [14,17,18]. However, research on the resistance to oxygen transport at intermediate temperatures is not well developed. In this paper, the limiting current density and electrochemical impedance of different percentage contents of oxygen (0.5%, 1%, 1.5%, 2%, 4%, 8%, 12%, 16%, and 21%) at different pressures (100 kpa, 150 kpa, and 200 kpa) at 80 °C (Stable state), 100 °C, and 120 °C (Unstable state) were measured to calculate the resistance to oxygen transport of each component of the MEA at intermediate temperatures. Meanwhile, based on the experimental results, a fuel cell model adapted to intermediate temperatures was established to comprehensively analyze the reasons leading to the change in oxygen transport resistance at intermediate temperatures.

## 2. Materials and Methods

**Figure 1:**
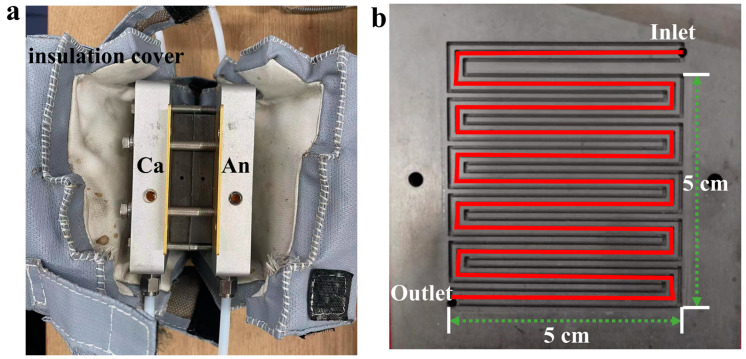
(**a**) Fixture used for fuel cell testing; (**b**) tri-serpentine flow field.

The PEM used in this study was nafion211. The Pt loading of the CL was CA/An@0.4/0.1 mg/cm^2^. A GDL with MPL was used in all experiments with the model number SGL22-BB. The assembled MEAs were mounted in a fixture with a 5 × 5 triple serpentine flow field, and the outside of the fixture was covered with an insulating sleeve to prevent the temperature from dissipating too quickly during the test, resulting in unstable cell performance. The fixture and flow field are shown in Figure 1. The cell assembly pressure was 5 MPa and the compression rate of the MEA was 20%. Experimental details and electrochemical measurements are recorded in the Supporting Information.

## 3. Theoretical Deduction

### 3.1. Cathodic Oxygen Transport Process

There are two transmission modes of gas after passing into the fuel cell: diffusion and convection. Convection mainly occurs in CH, and convection is affected by the size and shape of CH. The transmission of gas to the catalytic layer for reaction is mainly controlled by diffusion. To accurately assess the oxygen transmission resistance, it is necessary to maintain uniform gas concentration within the PEMFC. Consequently, high-flow rate and substantial gas flow are introduced into both the cathode and anode, ensuring minimal reaction consumption in comparison to the gas supplied to the cell. Simultaneously, because our PEMFC operates at an intermediate temperature, most of the water in the PEMFC will evaporate, and the large flow of gas can quickly take the water vapor away from the battery, keeping the internal humidity of the battery stable.

According to the equation derived by Baker et al., the total oxygen transfer resistance $R_{total}$ can be expressed as [8]:

(1) $$R_{total}=\frac{4F(p-p_{W})x_{O_{2}}^{dry-in}}{RTi_{lim}}$$

where *F* represents Faraday’s constant, p represents the gas pressure, $p_{W}$ is the water vapor pressure, $x_{O_{2}}^{dry-in}$ is the inlet concentration versus the dry mole fraction of oxygen *R* is the ideal gas constant, while *T* is the absolute cell temperature and $i_{lim}$ is the limiting current density.

Oxygen needs to be transported through CH, GDL, and CL in the PEMFC. Each of these stages introduces resistance to oxygen transport, so the total transmission resistance can be expressed as the sum of the resistance at each step:

(2) $$R_{total}=R_{CH}+R_{GDL}+R_{CL}$$

Among them, $R_{CH}$ represents the oxygen transmission resistance in the flow channel. Since our PEMFC operates in an intermediate temperature range when the battery reacts to produce water, the majority of the water will evaporate, and due to the large gas flow, the resistance of the oxygen in CH is very small, so we think $R_{CH}$ can be ignored.

$R_{GDL}$ is the transmission resistance of oxygen in GDL. GDL is mainly composed of GDB and MPL, so $R_{GDL}$ can be expressed as:

(3) $$R_{GDL}=R_{GDB}+R_{MPL}$$

In Formula (3), $R_{GDB}$ and $R_{MPL}$ represent the transmission resistance of oxygen in the GDB and microporous layer, respectively. The diffusion of oxygen in GDB is primarily governed by intermolecular diffusion, while oxygen transport in the MPL is primarily controlled by Knudsen diffusion.

$R_{CL}$ is the transmission resistance of oxygen in CL, which can be divided into the process of transmission within the pores of the CL and the process of transmission from the pores to the platinum surface. The former is Knudsen diffusion and the latter is local transmission, that is:

(4) $$R_{CL}=R_{CL,\;Knu}+R_{local}$$

The local mass transfer process of oxygen includes dissolution and diffusion processes at the Pt/C three-phase interface. The time required for its mass transfer τ is:

(5) $$\tau =\delta r_{local}$$

where δ is the equivalent thickness of the ionomer membrane within the catalytic layer, (of the order of 10^−8^ m) and $r_{local}$ is the local mass transfer resistance per unit Pt surface area of the order of 10^−3^ s m^−1^. Since the time required for this process is sufficiently short, the local transmission process can be considered a steady state.

**Figure 2:**
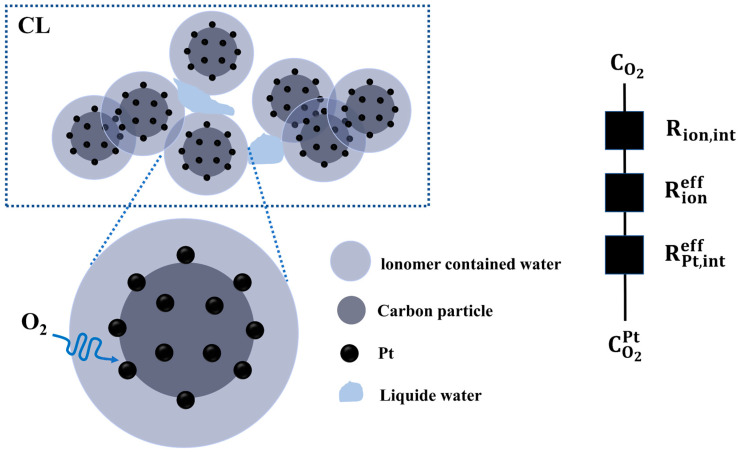
Schematic diagram of local transport of oxygen from the pores of the cathode catalytic layer to the Pt surface.

There are mainly three transmission resistances during the local transmission of oxygen in the cathode catalytic layer. These include the ionomer film interface transmission resistance ($R_{\mathrm{ion},\mathrm{int}}$), the intra-ion film diffusion resistance ($R_{ion}^{eff}$), and the interfacial transmission resistance near the platinum surface due to limited adsorption on the platinum surface ($R_{Pt,int}^{eff}$) [19,20] (the process is shown in Figure 2). Therefore, $r_{local}$ can be expressed by the following formula:

(6) $$r_{local}=R_{ion,int}+R_{ion}^{eff}+R_{Pt,int}^{eff}$$

$R_{ion,int}$ and $R_{ion}^{eff}$ can be represented as the interfacial transport resistance and effective diffusion resistance of ionic polymers, respectively. $R_{Pt,int}^{eff}$ is the effective interface transmission resistance of platinum, and their detailed derivation can be found in the supporting information.

(7) $$R_{ion,int}=k_{1}\frac{\delta_{ion}}{D_{O_{2},ion}}$$

(8) $$R_{ion}^{eff}=\frac{\delta_{ion}}{D_{O_{z},ion}}\frac{x\rho_{Pt}r_{Pt}{\left(r_{C}+\delta_{ion}\right)}^{2}(1-wt\%)}{\rho_{C}r_{C}^{3}wt\%(1-\theta_{PtOH})}$$

(9) $$R_{Pt,int}^{eff}=k_{2}\frac{\delta_{ion}}{D_{O_{z},ion}}\frac{x\rho_{Pt}r_{Pt}{\left(r_{C}+\delta_{ion}\right)}^{2}(1-wt\%)}{\rho_{C}r_{C}^{3}wt\%(1-\theta_{PtoH})}$$

The $R_{local}$ of the entire electrode can be calculated as:

(10) $$R_{local}=\frac{r_{local}}{m_{pt}a_{ECSA}}$$

where $m_{pt}$ is the loading amount of Pt, and $a_{ECSA}$ is the electrochemical active surface area (ECSA) of Pt particles.

Therefore, the resistance of the catalytic layer is finally:

(11) $$R_{CL}=R_{cl,Knu}+\frac{R_{ion,int}+R_{ion}^{eff}+R_{Pt,int}^{eff}}{m_{pt}a_{ECSA}}$$

The intermolecular diffusion resistance is proportional to the gas pressure, while the Knudsen diffusion resistance and the local mass transfer resistance of the catalytic layer are independent of pressure. We denote the pressure-dependent gas resistance as $R_{P}$ and the pressure-independent gas resistance as $R_{NP}$. Thus, we can obtain:

(12) $$R_{P}=K_{3}P$$

Among them, *K*_3_ is the intermolecular diffusion resistance coefficient of oxygen in the GDL substrate, while P is the pressure.

Therefore, the total gas transfer resistance $R_{total}$ can be simplified to:

(13) $$R_{total}=R_{P}+R_{NP}$$

(14) $$R_{total}=K_{3}P+R_{NP}$$

Because $R_{NP}$ is independent of pressure, *K* can be expressed as:

(15) $$K_{3}=\frac{dR_{total}}{dP}$$

Therefore, using pressure *P* versus $R_{total}$ to plot, $K_{3}$ is the slope of the curve between $R_{total}$ and pressure *P*. The intersection of the curve and the Y-axis is $R_{NP}$. Substituting the values into Formulas (7)–(10), yields the local transmission resistance, and subsequently, subtracting $R_{NP}$ from the local transmission resistance gives the Knudsen diffusion resistance.

### 3.2. Oxygen Transport Modeling

As an important complement to experiments, geometric and mathematical modeling has become a powerful tool for gaining insight into oxygen transport mechanisms. To improve the study of gas transmission within the fuel cell membrane electrode and promote visual research within the fuel cell, we developed a comprehensive geometric model that describes the fuel cell’s working process. We established corresponding mathematical models through different conservation equations by setting the source term and boundary conditions and making sufficient assumptions for the models.

#### 3.2.1. Geometric Model

**Figure 3:**
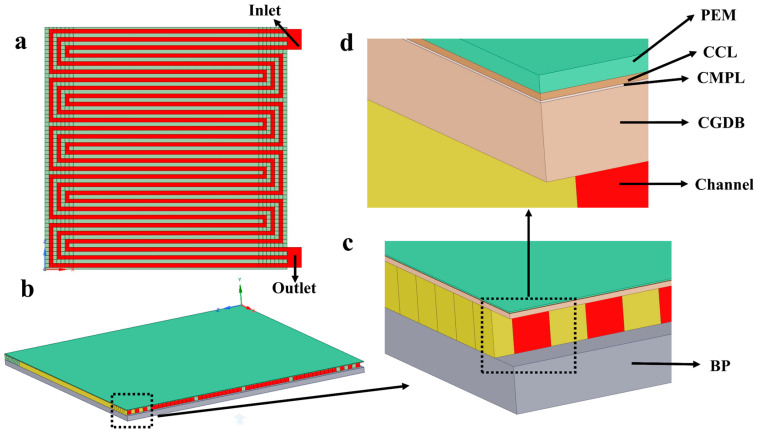
Geometrical model of fuel cell with triple serpentine flow channel (**a**) flow field; (**b**) cathode cross-section; (**c**,**d**) cathode cross-section magnification.

Figure 3 illustrates the fuel cell geometric model developed in this study and features three serpentine flow channels. The model includes the gas inlet and outlet of the cathode and anode, BP, CH, GDL, MPL, CL, and PEM. The specific parameters of the geometric model are shown in Table S1. Both the cathode and anode of this fuel cell model adopt three serpentine flow channels. Among them, the dashed box indicates that the subsequent figure is an enlarged detail view of a specific area highlighted in the previous figure.

#### 3.2.2. Mathematical Model

PEMFC is a complex system with multiple scales and multiple physical fields. Simulation and calculation analysis of PEMFC requires an accurate and clear understanding of its internal reaction. The control equations of the basic computational fluid model of fuel cells are as follows [21,22,23]:

The mass conservation equation of the mixed gas is shown in Equation (16):

(16) $$\frac{\partial \left[\varepsilon \rho_{g}\left(1-s_{lq}\right)\right]}{\partial t}+\nabla \cdot \left(\rho_{g}\mu_{g}\right)=S_{m}$$

where ε is the porosity, $\rho_{g}$ is the density of the mixed gas, $s_{lq}$ is the saturation of water, t is the time, $\mu_{g}$ is the gas flow rate, and $S_{m}$ is the mass source term.

(17) $$S_{m}=\left\{\begin{array}{c} 0\;\;\;\;\;\;\;\;\;\;\;\;\;\;\;\;\;\;\;\;\;\;\;\;\;\;\;\;\;\;\;\;\;\;\;\;\;\;\;\;\;\;\;\;\;\;\;\;\;\;\;BPs\\ S_{v}\;\;\;\;\;\;\;\;\;\;\;\;\;\;\;\;\;\;\;\;\;\;\;\;\;\;\;\;\;\;\;\;\;GDLs,MPLs\\ -\left(j_{a}/2F\right)M_{H_{2}}+S_{v}\;\;\;\;\;\;\;\;\;\;\;\;a-CLs\\ -\left(j_{c}/4F\right)M_{O_{2}}+S_{v}\;\;\;\;\;\;\;\;\;\;\;\;\;c-CLs\\ 0\;\;\;\;\;\;\;\;\;\;\;\;\;\;\;\;\;\;\;\;\;\;\;\;\;\;\;\;\;\;\;\;\;\;\;\;\;membranes \end{array}\right.$$

where $S_{v}$ is the mass source term of water vapor and j is the current density.

(18) $$S_{v}=\left\{\begin{array}{c} -S_{v-l}\;\;\;\;\;\;\;\;\;\;\;\;\;\;\;\;\;\;\;\;\;\;\;\;\;\;\;\;\;\;\;\;\;\;\;\;\;\;\;\;\;\;\;\;\;\;\;\;\;\;\;\;\;\;\;\;\;\;\;\;\;\;\;\;\;\;\;GDLs,MPLs\\ S_{n-v}M_{H_{2}O}-S_{v-l}\;\;\;\;\;\;\;\;\;\;\;\;\;\;\;\;\;\;\;\;\;\;\;\;\;\;\;\;\;\;\;\;\;\;\;\;\;\;\;\;\;\;\;\;\;\;\;\;\;\;\;\;\;\;\;\;a-CLs\\ S_{n-v}M_{H_{2}O}-S_{v-l}+\left(j_{c}/2F\right)M_{H_{2}O}\;\;\;\;\;\;\;\;\;\;\;\;\;\;\;\;\;\;\;\;\;\;\;\;\;\;\;\;c-CLs\\ S_{n-v}M_{H_{2}O}\;\;\;\;\;\;\;\;\;\;\;\;\;\;\;\;\;\;\;\;\;\;\;\;\;\;\;\;\;\;\;\;\;\;\;\;\;\;\;\;\;\;\;\;\;\;\;\;\;\;\;\;\;\;\;\;\;\;\;\;\;\;CL\;ionomer \end{array}\right.$$

where $S_{v-l}$ is the source term for the conversion of water vapor into liquid water, and $S_{n-v}$ is the source term for the conversion of membrane water into water vapor.

(19) $$S_{v-l}=\left\{\begin{array}{c} R_{cond}\varepsilon \left(1-s_{lq}\right)\frac{\left(P_{v}-P_{sat}\right)M_{H_{2}O}}{RT}\;\;\;\;\;\;\;\;\;\;\;\;\;\;\;\;\;\;P_{v}\geq P_{sat}\\ R_{cond}\varepsilon s_{lq}\frac{\left(P_{v}-P_{sat}\right)M_{H_{2}O}}{RT}\;\;\;\;\;\;\;\;\;\;\;\;\;\;\;\;\;\;\;\;\;\;\;\;\;\;\;\;\;\;\;P_{v}<P_{sat} \end{array}\right.$$

where $R_{cond}$ represents the rate of liquefaction phase change conversion, $P_{v}$ denotes the partial pressure of water vapor, and $P_{sat}$ is the saturated vapor pressure of water. The calculation formula is [24]:

(20) $$\begin{array}{l} {\mathrm{Log}}_{10}P_{sat}=-2.1794+0.02953\left(T-273.17\right)-9.1837\times 10^{-5}{\left(T-273.17\right)}^{2}\\ \;\;\;\;\;\;\;\;\;\;\;\;\;\;\;\;\;\;\;\;\;\;\;\;\;\;\;\;+1.4454\times 10^{-7}{\left(T-273.17\right)}^{3} \end{array}$$

(21) $$S_{n-v}=R_{n-v}\frac{\rho_{mem}}{EW}\left(\lambda_{nf}-\lambda_{eq}\right)\left(1-s_{lq}\right)$$

where $R_{n-v}$ is the phase change conversion rate of membrane water into water vapor, $\rho_{mem}$ is the density of PEM, EW is the equivalent mass, $\lambda_{nf}$ is the membrane water content, and $\lambda_{eq}$ is the equilibrium membrane water content [25].

(22) $$\lambda_{eq}=\left\{\begin{array}{l} 0.043+17.81a-39.85a^{2}+36.0a^{3}\;\;\;\;\;\;\;\;\;\;\;\;0\leq a\leq 1\\ 14.0+1.4\left(a-1\right)\;\;\;\;\;\;\;\;\;\;\;\;\;\;\;\;\;\;\;\;\;\;\;\;\;\;\;\;\;\;\;\;\;\;\;\;\;\;\;\;1<a\leq 3 \end{array}\right.$$

where a is the activity of water, and its calculation formula is [26]:

(23) $$a=\frac{P_{v}}{P_{sat}}+2s_{lq}$$

In porous media, for Newtonian fluids, the momentum equation can be expressed as:

(24) $$\frac{\partial \left(\rho \mu \right)}{\partial t}+\nabla \left(\rho \mu \cdot \frac{\mu }{\varepsilon }\right)=-\nabla \left(\varepsilon P\right)+\nabla \cdot \left(\mu \nabla \mu \right)-\varepsilon \frac{\mu }{K}\mu +\varepsilon \rho g$$

where μ is the dynamic viscosity and μ is the flow rate.

The composition conservation equation is shown in Equation (25):

(25) $$\frac{\partial \left[\varepsilon \rho_{j}\left(1-s_{lq}\right)\right]}{\partial t}+\nabla \cdot \left(\rho_{j}\mu_{g}\right)=\nabla \cdot \left(D_{j}^{eff}\nabla \rho_{j}\right)+S_{j}$$

where j represents any component (hydrogen, oxygen, and various forms of water), $S_{j}$ is the mass source term of component j, and $D_{j}^{eff}$ is the effective diffusion coefficient of component j.

(26) $$D_{j}^{eff}=D_{j}\varepsilon^{1.5}{\left(1-s_{lq}\right)}^{1.5}$$

where $D_{j}$ is the intrinsic diffusion coefficient of component j. The intrinsic diffusion coefficient of each component is as follows [27]:

(27) $$\begin{array}{c} D_{H_{2}}=1.005\times {\left(T/333.15\right)}^{1.5}\left(101325/P\right)\\ D_{v}^{a}=1.005\times {\left(T/333.15\right)}^{1.5}\left(101325/P\right)\\ D_{O_{2}}=0.2652\times {\left(T/333.15\right)}^{1.5}\left(101325/P\right)\\ D_{v}^{c}=0.2982\times {\left(T/333.15\right)}^{1.5}\left(101325/P\right) \end{array}$$

where $D_{H_{2}}$, $D_{v}^{a}$, $D_{O_{2}}$ and $D_{v}^{c}$ represent the hydrogen diffusion coefficient, anode water vapor diffusion coefficient, oxygen diffusion coefficient, and cathode water vapor diffusion number, respectively.

(28) $$S_{lq}=\left\{\begin{array}{c} S_{v-l}\;\;\;\;\;\;\;\;\;\;\;\;\;\;\;\;\;\;\;\;\;\;\;\;\;\;\;\;\;\;\;\;\;\;\;\;\;\;\;\;\;\;\;\;\;\;\;\;GDLs,MPLs\\ \;S_{v-l}\;\;\;\;\;\;\;\;\;\;\;\;\;\;\;\;\;\;\;\;\;\;\;\;\;\;\;\;\;\;\;\;\;\;\;\;\;\;\;\;\;\;\;\;\;\;\;\;\;\;\;\;\;\;\;\;a-CLs\\ S_{v-l}\;\;\;\;\;\;\;\;\;\;\;\;\;\;\;\;\;\;\;\;\;\;\;\;\;\;\;\;\;\;\;\;\;\;\;\;\;\;\;\;\;\;\;\;\;\;\;\;\;\;\;\;\;\;\;\;\;c-CLs\\ 0\;\;\;\;\;\;\;\;\;\;\;\;\;\;\;\;\;\;\;\;\;\;\;\;\;\;\;\;\;\;\;\;\;\;\;\;\;\;\;\;\;\;\;\;\;\;\;\;\;\;\;\;\;\;\;\;\;CL\;ionomer \end{array}\right.$$

(29) $$S_{nf}=\left\{\begin{array}{c} 0\;\;\;\;\;\;\;\;\;\;\;\;\;\;\;\;\;\;\;\;\;\;\;\;\;\;\;\;\;\;\;\;\;\;\;\;\;\;\;\;\;\;\;\;\;\;\;\;\;\;\;\;\;\;\;\;\;\;\;\;\;\;\;\;\;\;\;\;\;\;\;\;\;\;\;\;\;\;\;\;\;\;\;\;GDLs,MPLs\\ 0\;\;\;\;\;\;\;\;\;\;\;\;\;\;\;\;\;\;\;\;\;\;\;\;\;\;\;\;\;\;\;\;\;\;\;\;\;\;\;\;\;\;\;\;\;\;\;\;\;\;\;\;\;\;\;\;\;\;\;\;\;\;\;\;\;\;\;\;\;\;\;\;\;\;\;\;\;\;\;\;\;\;\;\;\;\;\;\;\;\;\;\;a-CLs\\ 0\;\;\;\;\;\;\;\;\;\;\;\;\;\;\;\;\;\;\;\;\;\;\;\;\;\;\;\;\;\;\;\;\;\;\;\;\;\;\;\;\;\;\;\;\;\;\;\;\;\;\;\;\;\;\;\;\;\;\;\;\;\;\;\;\;\;\;\;\;\;\;\;\;\;\;\;\;\;\;\;\;\;\;\;\;\;\;\;\;\;\;\;c-CLs\\ -S_{n-v}M_{H_{2}O}+\nabla \left[\left(\lambda_{nf}/8F\right)\kappa_{ion}^{eff}\nabla \varphi_{ele}\right]M_{H_{2}O}\;\;\;\;\;\;\;\;\;\;\;\;\;\;\;\;\;CL\;ionomer \end{array}\right.$$

where $\varphi_{ele}$ is the electron potential, $\kappa_{ion}^{eff}$ is the effective ion conductivity, and its expression is:

(30) $$\kappa_{ion}^{eff}=\omega^{1.5}\kappa_{ion}$$

where ω is the ionomer volume fraction, $\kappa_{ion}$ is the ionic conductivity, and its expression is [25]:

(31) $$\kappa_{ion}=\left(0.5139\lambda_{nf}-0.326\right)exp\left[1268\left(\frac{1}{303.15}-\frac{1}{T}\right)\right]\;\;\;\left(S\cdot m^{-1}\right)$$

(32) $$S_{H_{2}}=-\frac{j_{a}}{2F}M_{H_{2}}$$

(33) $$S_{O_{2}}=-\frac{j_{c}}{4F}M_{O_{2}}$$

(34) $$S_{H_{2}O}=j\left(\frac{n_{d}}{F}+\frac{M_{H_{2}O}}{2F}\right)$$

The source terms in Equations (32)–(34) are the consumption/production rates of hydrogen, oxygen, and water vapor. Here, $M_{H_{2}}$, $M_{O_{2}}$ and $M_{H_{2}O}$ represent the molecular weights of hydrogen, oxygen, and water, respectively. The net resistance coefficient, denoted as $n_{d}$, is calculated using the following formula [26]:

(35) $$n_{d}=\frac{2.5\lambda }{22}$$

The liquid water conservation equation is applied in CLs, MPLs, and GDLs, as shown in Equation (36).

(36) $$\frac{\partial \left(\varepsilon s_{lq}\rho_{lq}\right)}{\partial t}=\nabla \cdot \left(-\frac{K_{lq}}{\mu_{lq}}\frac{dP_{c}}{ds_{lq}}\rho_{lq}\nabla s_{lq}\right)+S_{lq}$$

where $K_{lq}$ is the effective permeability of liquid water, $\mu_{lq}$ is the viscosity of liquid water, and $P_{c}$ is the capillary pressure.

(37) $$P_{c}=\left\{\begin{array}{c} \sigma \cos\theta {\left(\varepsilon /K_{0}\right)}^{0.5}\times \\ \left[1.42\left(1-s_{lq}\right)-2.12{\left(1-s_{lq}\right)}^{2}+1.26{\left(1-s_{lq}\right)}^{3}\right]\;\;\theta <90{}^{\circ}\\ \sigma \cos\theta {\left(\varepsilon /K_{0}\right)}^{0.5}\times \\ \left[1.42s_{lq}-2.12{s_{lq}}^{2}+1.26{s_{lq}}^{3}\right]\;\;\;\;\;\;\;\;\;\;\;\;\;\;\;\;\;\;\;\;\;\;\;\;\;\;\;\;\;\;\;\;\;\;\;\;\;\theta >90{}^{\circ} \end{array}\right.$$

where σ is the surface tension of liquid water, θ is the contact angle, and $K_{0}$ is the absolute permeability.

(38) $$K_{lq}=K_{0}{s_{lq}}^{4}$$

(39) $$\mu_{lq}=2.414\times 10^{-5}\times 10^{247.8/\left(T-140\right)}\;\;\;\left(kg\cdot m^{-1}\cdot s^{-1}\right)$$

The conservation equation of unfrozen membrane water is shown in Equation (40)

(40) $$\frac{\partial }{\partial t}\left(\frac{\rho_{mem}\omega \lambda_{nf}}{EW}\right)+\nabla \left(\frac{2.5}{22F}\kappa_{ion}^{eff}\nabla \varphi_{ele}\lambda_{nf}\right)=\nabla \left(\omega^{1.5}\frac{\rho_{mem}}{EW}D_{d}^{eff}\nabla \lambda_{nf}\right)+S_{nf}$$

where $D_{d}^{eff}$ is the effective diffusion coefficient of membrane water, and its expression is [27]:

(41) $$D_{d}^{eff}=\left\{\begin{array}{c} 3.1\times 10^{-7}\lambda_{nf}\\ \left[exp\left(0.28\lambda_{nf}\right)-1\right]exp\left(-2346/T\right)\;\;\;\;\;\;\;\;\;\;\;\;\;\;\;\;0<\lambda_{nf}\leq 3\\ 4.17\times 10^{-8}\lambda_{nf}\\ \left[161exp\left(-\lambda_{nf}\right)+1\right]exp\left(-2346/T\right)\;\;\;\;\;\;\;\;\;\;\;\;3<\lambda_{nf}\leq 17\\ 4.1\times 10^{-10}{\left(\lambda_{nf}/25\right)}^{0.15}\\ \left[1+\tanh\left(\left(\lambda_{nf}-2.5\right)/1.4\right)\right]\;\;\;\;\;\;\;\;\;\;\;\;\;\;\;\;\;\;\;\;\;\;\;\;\;\;\;\;\;\;\;\lambda_{nf}>17 \end{array}\right.$$

The energy conservation equation is shown in Equation (42):

(42) $$\begin{array}{l} \frac{\partial }{\partial t}\left(\varepsilon s_{lq}\rho_{lq}C_{p,lq}T+\varepsilon \left(1-s_{lq}\right)\rho_{g}C_{p,g}T\right)\\ \;\;\;\;\;\;\;\;\;\;\;\;\;\;\;\;\;\;\;\;\;\;\;\;\;\;\;+\nabla \left(\varepsilon s_{lq}\rho_{lq}C_{p,lq}\mu_{lq}T+\varepsilon \left(1-s_{lq}\right)\rho_{g}C_{p,g}\mu_{lq}T\right)\\ \;\;\;\;\;\;\;\;\;\;\;\;\;\;\;\;\;\;\;\;\;\;\;\;\;\;\;=\nabla \left(\kappa^{eff}\nabla T\right)+S_{T} \end{array}$$

where $C_{p}$ is the molar heat capacity at constant pressure and $\kappa^{eff}$ is the effective thermal conductivity.

(43) $$S_{T}=\left\{\begin{array}{l} {\left\|\nabla \varphi_{ele}\right\|}^{2}\kappa_{ele}^{eff}\;\;\;\;\;\;\;\;\;\;\;\;\;\;\;\;\;\;\;\;\;\;\;\;\;\;\;\;\;\;\;\;\;\;\;\;\;\;\;\;\;\;\;\;\;\;\;\;\;\;\;\;\;\;\;\;\;\;\;\;\;\;\;\;\;\;\;\;\;\;\;\;\;\;\;\;\;\;\;\;\;\;BPs\\ {\left\|\nabla \varphi_{ele}\right\|}^{2}\kappa_{ele}^{eff}+h_{v-l}S_{v-l}\;\;\;\;\;\;\;\;\;\;\;\;\;\;\;\;\;\;\;\;\;\;\;\;\;\;\;\;\;\;\;\;\;\;\;\;\;\;\;\;\;\;\;\;\;\;\;\;\;GDLs,MPLs\\ \;\;\;\;\;\;\;\;\;\;j_{a}\left|\eta_{a}\right|+{\left\|\nabla \varphi_{ele}\right\|}^{2}\kappa_{ele}^{eff}+{\left\|\nabla \varphi_{ion}\right\|}^{2}\kappa_{ion}^{eff}+\frac{j_{a}T∆S_{a}}{2F}\\ +h_{v-l}S_{v-l}-h_{n-v}S_{n-v}M_{H_{2}O}\;\;\;\;\;\;\;\;\;\;\;\;\;\;\;\;\;\;\;\;\;\;\;\;\;\;\;\;\;\;\;\;\;\;\;\;\;\;\;\;\;\;\;\;\;\;\;\;a-CLs\\ \;\;\;\;\;\;\;\;\;\;j_{c}\left|\eta_{c}\right|+{\left\|\nabla \varphi_{ele}\right\|}^{2}\kappa_{ele}^{eff}+{\left\|\nabla \varphi_{ion}\right\|}^{2}\kappa_{ion}^{eff}+\frac{j_{c}T∆S_{c}}{2F}\\ +h_{v-l}S_{v-l}-h_{n-v}S_{n-v}M_{H_{2}O}\;\;\;\;\;\;\;\;\;\;\;\;\;\;\;\;\;\;\;\;\;\;\;\;\;\;\;\;\;\;\;\;\;\;\;\;\;\;\;\;\;\;\;\;\;\;\;\;c-CLs\\ {\left\|\nabla \varphi_{ion}\right\|}^{2}\kappa_{ion}^{eff}+h_{n-f}S_{n-f}M_{H_{2}O}\;\;\;\;\;\;\;\;\;\;\;\;\;\;\;\;\;\;\;\;\;\;\;\;\;\;\;\;\;\;\;\;\;\;\;\;\;\;\;\;membranes \end{array}\right.$$

where $\kappa_{ele}^{eff}$ is the effective electronic conductivity, h is the phase change generation enthalpy, $\kappa_{ion}^{eff}$ is the effective ion conductivity, ∆S is the entropy change, and η is the electrode potential. The relationship among electrode potential, proton potential, and electron potential is as follows [28]:

(44) $$\eta_{a}=\varphi_{ele}-\varphi_{ion}$$

(45) $$\eta_{c}=\varphi_{ele}-\varphi_{ion}-U_{0}$$

(46) $$U_{0}=1.23-0.9\times 10^{-3}(T-298)$$

The proton conservation equation is shown in Equations (47) and (48):

(47) $$\nabla \cdot \left(\kappa_{ion}^{eff}\nabla \varphi_{ion}\right)+S_{ion}=0$$

(48) $$S_{ion}=\left\{\begin{array}{c} j_{a}\;\;\;\;\;\;\;\;\;\;\;\;\;\;\;a-CLs\\ -j_{c}\;\;\;\;\;\;\;\;\;\;\;\;c-CLs \end{array}\right.$$

The electron conservation equations are shown in Equations (49) and (50):

(49) $$\nabla \cdot \left(\kappa_{ele}^{eff}\nabla \varphi_{ele}\right)+S_{ele}=0$$

(50) $$S_{ele}=\left\{\begin{array}{c} {-j}_{a}\;\;\;\;\;\;\;\;\;\;\;\;\;\;\;a-CLs\\ j_{c}\;\;\;\;\;\;\;\;\;\;\;\;\;\;\;\;\;\;\;c-CLs \end{array}\right.$$

#### 3.2.3. Model Assumptions

This study established a three-dimensional steady-state fuel cell model. Considering the accuracy of the model, we constructed and solved the entire model based on the following assumptions:

(1) The fuel cell operating environment is steady state;
(2) All gases involved in this study are considered ideal gases;
(3) Both GDL and CL are porous media with uniform porosity;
(4) There is no hydrogen permeation in PEM, and only the conduction of protons and hydronium ions in the membrane is considered.

The boundary conditions of the model, the solution method, the meshing of the model, and the validation of the model will be described in detail in the support information.

## 4. Discussion

### 4.1. Calculation and Analysis of the Resistance of Each Part

**Figure 4:**
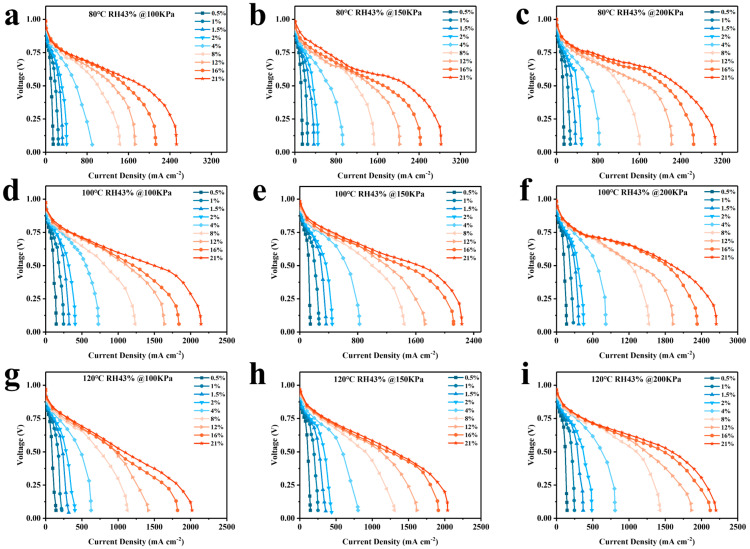
Polarization curves of fuel cells using different percentage contents of oxygen at different temperatures and back pressures. (**a**) 80 °C, 100 kpa, (**b**) 80 °C, 150 kpa, (**c**) 80 °C, 200 kpa, (**d**) 100 °C, 100 kpa, (**e**) 100 °C, 150 kpa, (**f**) 100 °C, 200 kpa, (**g**) 120 °C, 100 kpa, (**h**) 120 °C 150 kpa, (**i**) 120 °C, 200 kpa.

Figure 4 illustrates the polarization profiles at different temperatures and pressures. The limiting current occurs at the late stage of the concentrated polarization of the cell for all conditions, and all curves reach the maximum current density when the voltage reaches about 0.2 V. The experimental results are affected when the voltage drops to about 0.1 V because hydrogen evolution occurs at the cathode catalyst [29]. Therefore, the current density at 0.2 V is considered the limiting current density.

**Figure 5:**
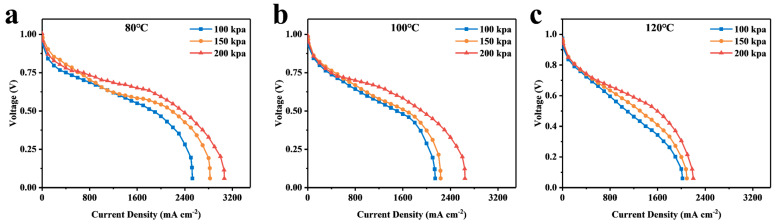
Polarization curves of the fuel cell at 21% oxygen content for different temperature and back pressure conditions. (**a**) 80 °C; (**b**) 100 °C; (**c**) 120 °C.

The curves at 21% oxygen concentration (simulating conditions of the cathode as air) are depicted separately in Figure 5, which shows that, at temperatures of 80 °C, 100 °C, and 120 °C, increasing the back pressure enhances the cell’s limit current density at all temperatures, indicating that an increase in back pressure can improve cell performance. According to Henry’s Law, the solubility of gases (hydrogen and oxygen) in the electrolyte increases with increasing pressure. In fuel cells, this means that more reactive gases are available for electrochemical reactions within the electrolyte, thereby enhancing efficiency. Furthermore, as pressure increases, the concentration of hydrogen and oxygen near the catalyst surface also increases, which accelerates the reaction rate and improves overall performance.

**Figure 6:**
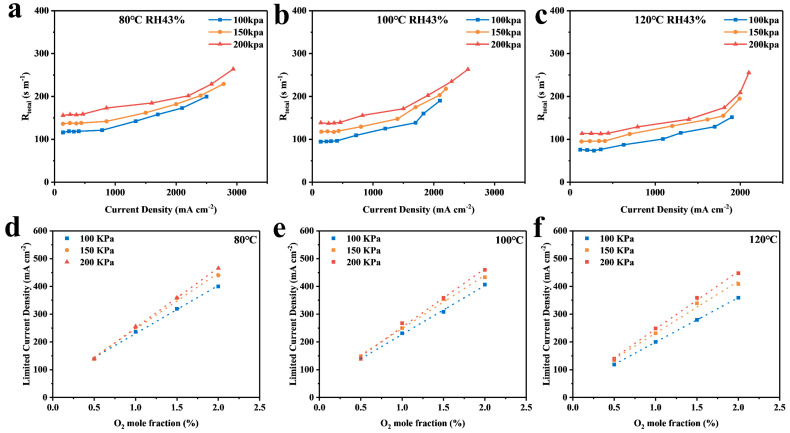
(**a**–**c**) Plots of ultimate current density versus total oxygen transport resistance for 0.5–21% oxygen content at 80 °C, 100 °C, and 120 °C; (**d**–**f**) Plots of 0.5–2% oxygen content versus ultimate current density at 80 °C, 100 °C, and 120 °C.

The calculation of $R_{total}$ was based on the measurement of the limiting current density and the use of Equation (1). The obtained results were then plotted against the total oxygen transport resistance for limiting current densities with varying oxygen content percentages, as illustrated in Figure 6a–c.

Figure 6a–c demonstrate that the total oxygen transfer resistance of the cell remains almost unchanged when less than 2% oxygen content is used. This suggests that the cell is in a dry state internally, producing less water from the reaction, resulting in negligible resistance to oxygen transfer. At this point, the intrinsic properties of MEA electrodes determine the oxygen transfer [11], and, as the oxygen content increases, the water produced by the ORR reaction also increases. Despite using a large airflow to remove the water from the cell, a small amount of water remains in the CL and GDL, which affects gas transfer and causes a rapid increase in total oxygen transfer resistance.

**Figure 7:**
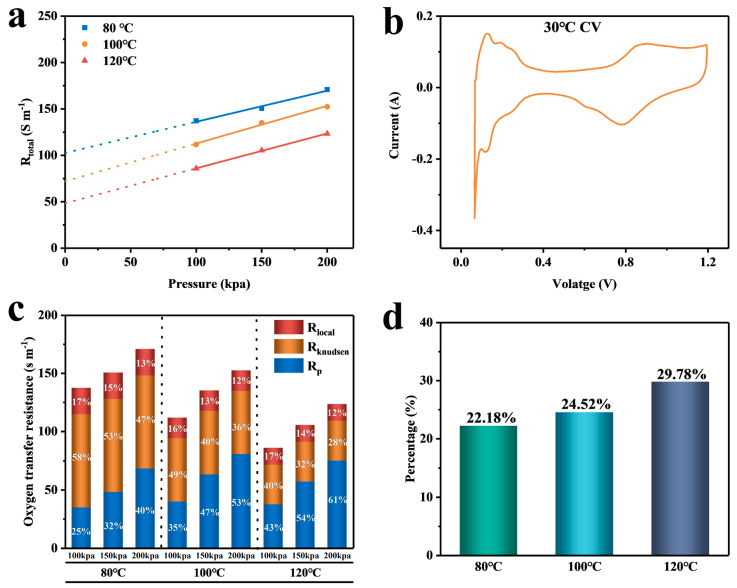
(**a**) Plot of total oxygen transport resistance versus total pressure; (**b**) CV curve at 30 °C; (**c**) percentage of resistance in each part of the MEA under different temperature pressures; (**d**) percentage of localized transport resistance at 80–120 °C under R_NP_.

Although $R_{total}$ can show differences in cell resistance under different intermediate temperature conditions, it cannot determine which part of the cell’s interior causes the change. To further study the mechanism of oxygen transport in the cell at intermediate temperatures, we plotted the limiting current in the dry state against the oxygen concentration, as shown in Figure 6d–f. The slope of the graph is I_lim_/X_o2_, and the inverse of the slope is brought into Equation (1) to find the $R_{total}$ of the MEA, which is plotted against the back pressure (as shown in Figure 7a).

Figure 7a shows that $R_{total}$ decreases gradually as the pressure is reduced. At 0 kpa, $R_{total}$ equals R_NP_. To measure the electrode’s ECSA, we obtained the cell’s CV test (Figure 7b) by calculating the integral area of the hydrogen adsorption peak of the curve. The localized transport resistance of the CL at each temperature was calculated by inputting the calculated values into Equation (10). The resulting values are presented in Table 1 and visualized as a bar graph in Figure 7c. The figure shows that $R_{total}$ increases with increasing pressure at all three temperatures due to the increase in gas pressure inside the cell leading to an increase in the number of N_2_ molecules surrounding the O_2_ molecules per unit volume. As a result, the mean free range between the gas molecules decreases [18], making it more difficult for the molecules to reach the active site. This, in turn, increases the total oxygen transport resistance. Under the same pressure, increasing the temperature from 80 °C to 120 °C accelerates the reactivity of molecules. Additionally, the increase in temperature causes water to evaporate into water vapor more easily, resulting in less residual water in the CL and diffusion layer. This makes oxygen transfer easier, reducing the total oxygen transfer resistance. Figure 7d shows the percentage of $R_{local}$ to the whole pressure-independent resistance at different temperatures. From the figure, it can be seen that the percentage of $R_{local}$ increases with increasing temperature, which is 29.78% at 120 °C, which is 34% more than the percentage at 80 °C. At intermediate temperatures, local transport plays a significant role in the oxygen transport process due to the accelerated Ostwald ripening, which leads to the growth of platinum nanoparticles. As a result, the effective oxygen diffusion paths through the ionomers and the aqueous film are significantly increased [1,30,31], hindering gas transport and causing an increase in $R_{local}$. This, in turn, leads to a larger oxygen consumption and a decrease in cell voltage.

The Arrhenius equation states that the ORR rate increases by a factor of 3.6 when the temperature is raised from 80 °C to 120 °C [32]. Additionally, as temperature increases, $R_{total}$ decreases, resulting in an increase in the current density of the cell at the same voltage. Increasing the cell temperature surprisingly does not result in an increase in current density (Figure 4). This may be due to the fact that the increase in temperature accelerates the rate of the ORR reaction while simultaneously decreasing the equilibrium potential (E_0_) of the reaction by about 33 mV [33]. Butori et al. [18] corrected the measurements for changes in equilibrium potential. The current density at constant cathodic overpotential increased with increasing temperature. It can therefore be assumed that the increase in reaction kinetics by raising the temperature is offset by a decrease in the equilibrium potential, which does not increase current density.

**Figure 8:**
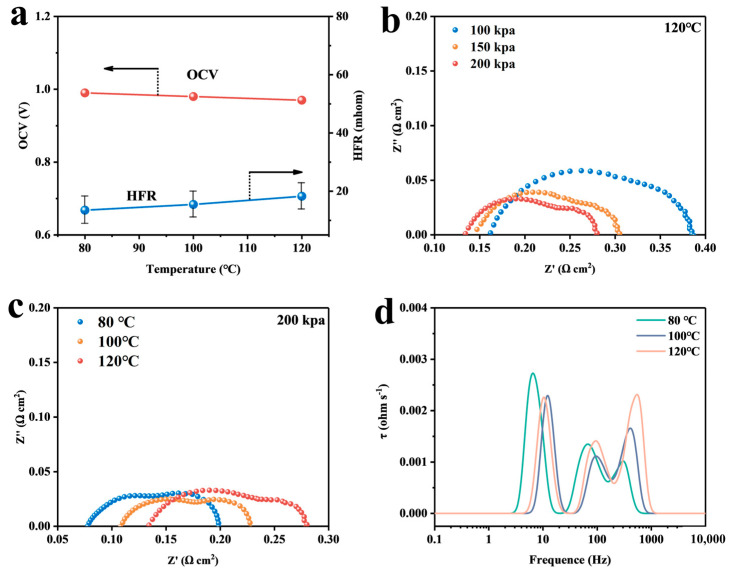
(**a**) OCV and HFR at the intermediate temperature of 100 kpa; (**b**) EIS at 120 °C for different pressures; (**c**) EIS at 200 kpa for different temperatures; and (**d**) DRT conversion plot for EIS at 200 kpa.

The open-circuit voltage (OCV) decreased from 0.99 V at 80 °C to 0.97 V at 120 °C as temperature increased (Figure 8a). This decrease was attributed to the higher gas crossover rate of the MEA membranes at higher temperatures. This led to the oxidation of hydrogen around the interface of the two electrodes between the electrolyte membrane and the catalyst layer, generating a mixed potential that decreased the OCV at intermediate temperatures [34,35,36]. Additionally, the mean value of HFR increased continuously with increasing temperature in agreement with previous studies [14,37].

The electrochemical impedance spectroscopy (EIS) technique was used to analyze the electrochemical phenomena that occur during cell operation to determine the resistance of each component that contributes to voltage loss. Figure 8b shows the Nyquist plot at 120 °C, revealing two distinct semicircles in all three curves. As back pressure increases, the resistance to charge transfer and material transport both decrease to varying degrees. Figure 8c displays the EIS for different temperatures at 200 kPa, where the HFR increases as the temperature rises. This is consistent with the data obtained through the limiting current. The decrease in proton conductivity is due to the reduction in water content in the proton exchange membrane state after an increase in temperature.

The distribution of relaxation time (DRT) technique is a relatively new analytical tool that can be used to extract relaxation time distributions in electrochemical systems through deconvolution. The deconvolution is performed through theoretical calculations to obtain the impedance of each part of the system rather than relying on a priori assumptions [38,39,40,41]. The EIS data were imported into MatLab and processed using a specific program to obtain the DRT image. The DRT plot displays substance transport impedance, charge transport impedance, and internal resistance as low, medium, and high frequencies, respectively. The heights of the peaks indicate the relative strengths of the impedances, and the value of the peaks integrated over the coordinate axes represents the magnitude of the impedance. As depicted in Figure 8d, the substance transport impedance decreases continuously as the temperature rises from 80 °C to 120 °C. This indicates a decrease in oxygen transport resistance, which is consistent with the calculation results presented in the previous section. Additionally, the intensity of the peak in the high-frequency region exhibits an opposite trend to that of the low frequency.

In addition, the reproducibility of the polarization curve and impedance characteristics of the Nafion211 film at temperatures of up to 100 °C was studied, and these data are provided in the Supplementary Material.

### 4.2. Analysis of Simulation Results

**Figure 9:**
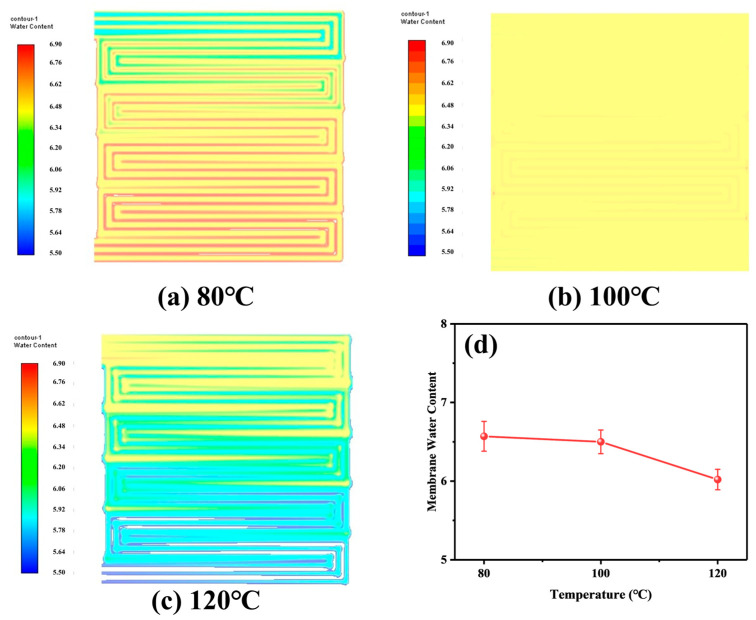
Water content in the film state at different temperatures (**a**) 80 °C; (**b**) 100 °C; (**c**) 120 °C; (**d**) specific values.

In membrane electrodes, the transfer of water and proton coupling is crucial, particularly when operating at intermediate temperatures. The diffusion of water in the membrane is more important than the transfer of protons, as changes in water content affect proton transfer in the membrane, which, in turn, affects the ORR reaction rate and ultimately causes fluctuations in cell performance. The ratio of water molecules to sulfonate ions in the MEA can be expressed in terms of water in the membrane state. In Figure 9, (a)–(c) is the nephogram of water content in the film state, (d) is the numeri-cal value, and the error bars represent the maximum and minimum values, which shows that as the temperature increases, water produced by the cathode ORR reaction rapidly evaporates into water vapor. Additionally, the large gas flow rate reduces the concentration diffusion of water, leading to a significant decrease in the water content of the membrane state inside the cell.

**Figure 10:**
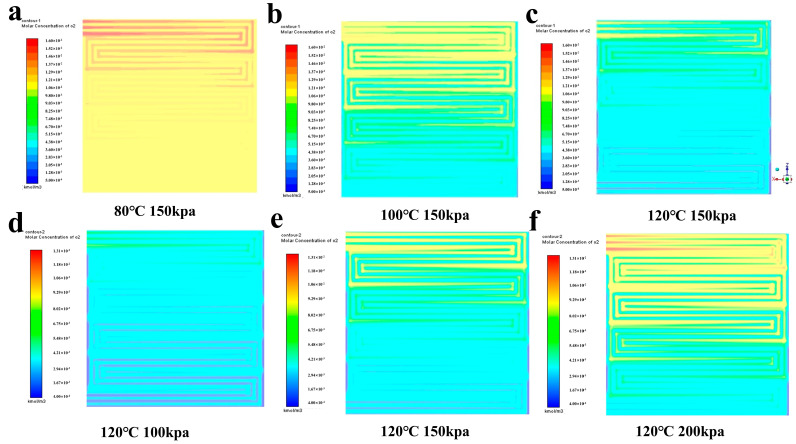
Molar concentration distribution of oxygen at the cathode.

To examine the impact of oxygen distribution in the cathode CL on performance, we simulated the distribution of oxygen molar concentration at half the thickness of the cathode CL under various conditions, and the results are presented in Figure 10. The gas molar concentration gradually decreases from the inlet to the outlet in the gas flow path, which may be attributed to the pressure difference induced by the flow field. The reaction process in the proton exchange membrane fuel cell may be affected due to the uneven distribution of oxygen, which can result in incomplete utilization of some reactants.

The concentration of oxygen at the cathode decreases with increasing temperature due to the increase in water vapor partial pressure. Figure 10a–c shows that, for the same back pressure, the molar concentration of oxygen at 120 °C is more than one order of magnitude lower than that at 80 °C. This reduction greatly decreases the concentration diffusion of oxygen at the cathode, leading to cell polarization. The concentrated differential polarization increase results in a decrease in cell voltage, which affects overall performance. Figure 10d–f shows that at 120 °C, increasing back pressure helps regulate gas flow conditions in the cell, slowing the rate of decrease in oxygen molar concentration and mitigating the effect of concentration-differential polarization. This strategy has the potential to improve the homogeneity of oxygen distribution and prevent performance degradation caused by temperature increases.

**Figure 11:**
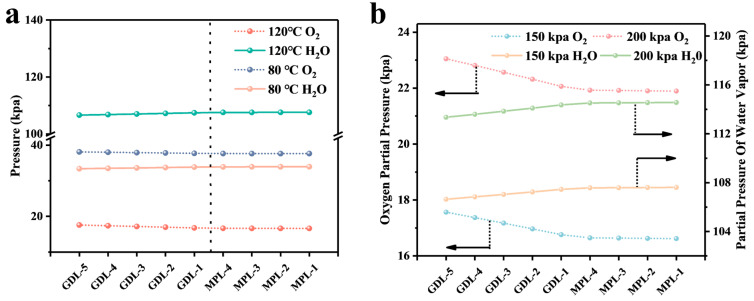
(**a**) Comparison of partial pressures of oxygen and water vapor at 80 °C and 120 °C at 150 kpa; (**b**) partial pressures of oxygen and water vapor at different pressures at 120 °C. (The pointing arrow represents the vertical axis corresponding to this data).

In order to better study the distribution of oxygen and water vapor partial pressures in CL and GDL at intermediate temperatures, MPL and GDB were divided into four and five equal parts, respectively, along the Y-axis direction, with smaller numbers indicating proximity to the CL. The partial pressures of oxygen and water vapor were calculated at each cross-section, as shown in Figure 11a. At 150 kPa, when the temperature increased from 80 °C to 120 °C, the partial pressure of water vapor increased significantly from 33 kPa to 107 kPa, while the partial pressure of oxygen decreased from 38 kPa to 17 kPa, a decrease of 55.3%. This can significantly impact the performance of the cell. When the back pressure was increased to 200 kPa, the oxygen partial pressure significantly improved to 22 kPa, as shown in Figure 11b. This improvement will accelerate the cathode ORR reaction rate and enhance the cell’s performance. The results suggest that higher temperatures require higher back pressure to ensure optimal cell performance.

## 5. Conclusions

In this study, the resistance of each part within the MEA was calculated more accurately from the limiting current densities obtained experimentally at different percentage contents of oxygen. The results showed that the total oxygen transport resistance (R_total_) was reduced by 27.8–37.5% at intermediate temperatures, with a gradual increase in temperature also being present. However, a significant increase in the effective oxygen diffusion paths of the ionomer and aqueous membranes due to Ostwald ripening increased the local transport resistance (R_local_) of the cathode CL as a percentage of the pressure-independent ground resistance by 34%, indicating that the effect of the R_local_ on cathode oxygen transport increased at intermediate temperatures. By analyzing the established fuel cell model at intermediate temperatures, it was found that, when the temperature was increased from 80 °C to 120 °C at a constant back pressure of 150 kpa, the water content in the membrane state inside the cell decreased significantly, and the partial pressure of water vapor increased drastically from 33 kpa to 107 kpa, whereas the partial pressure of oxygen decreased from 38 kpa to 17 kpa. Additionally, when this pressure was increased to 200 kpa, the partial pressure of oxygen rose to 22 kpa, meaning that the decreasing trend of oxygen partial pressure can be effectively alleviated by increasing the back pressure to ensure the performance of the cell. This study provides experimental and modeling ideas for the subsequent study of the oxygen transport mechanism in intermediate-temperature fuel cells.

## Figures and Tables

**Table 1:** Values of resistance of each component at different temperatures.

Temperature	Pressure	$R_{total}$ (s m^−1^)	$R_{NP}$ (s m^−1^)	$R_{local}$ (s m^−1^)
80 °C	100 kpa	137.3	102.63	22.76
	150 kpa	150.56	102.63	22.76
	200 kpa	170.8	102.63	22.76
100 °C	100 kpa	111.68	72.03	17.66
	150 kpa	135.11	72.03	17.66
	200 kpa	152.36	72.03	17.66
120 °C	100 kpa	85.75	48.46	14.43
	150 kpa	105.33	48.46	14.43
	200 kpa	123.3	48.46	14.43

## Data Availability

The original contributions presented in the study are included in the article/supplementary material, further inquiries can be directed to the corresponding author/s.

## Supplementary Materials

The following supporting information can be downloaded at: https://www.mdpi.com/article/10.3390/membranes14040072/s1, Figure S1: Comparison of simulated and experimental data; Figure S2. (a) Polarization curves of the cell at 80 °C, 100 °C and 120 °C before and after operation at 120 °C; (b) EIS at 80 °C, 100 °C and 120 °C before and after operation of the cell at °C; (c) Experienced LSV before and after 120 °C, at 80 °C; (d) Experienced LSV before and after 120 °C, at 100 °C; Table S1 Geometrical parameters of the fuel cell structure with triple serpentine flow channels [42,43].

## Abbreviations

**List of Symbols**			
$A_{\mathrm{ion}}^{\mathrm{eff}}$	The effective surface area of ionomer film per unit area of MEA	m^2^	
$A_{\mathrm{Pt}}^{\mathrm{eff}}$	Effective platinum surface area	m^2^	
a	Water activity		
$a_{ECSA}$	Electrochemical active area of platinum	cm^2^ g^−1^	
$C_{p}$	molar heat capacity at constant pressure	J K^−1^ mol^−1^	
$D_{O_{2},ion}$	The diffusion rate of oxygen in polymer membranes	m^2^ s^−1^	8.45 × 10^−10^
$E_{r}$	Thermodynamic reversible potential	V	
EW	Equivalent weight of the membrane	g mol^−1^	1000
F	Faraday’s constant	C mol^−1^	96,485
h	Enthalpy of phase transition formation	J mol^−1^	44.9
i	Current density	mA cm^2^	
k_1_	Interface resistance constant on the surface of ionomer films		8.5
k_2_	Interface resistance constant on platinum surface		16
M	Molecular weight	g mol^−1^	
$m_{Pt}$	Platinum loading in the cathode catalytic layer	mg cm^−2^	0.4
$N_{O}$	The molar flux of oxygen	mol s^−1^ m^2^	
$n_{Pt}$	The number of platinum particles in Pt/C		
$n_{d}$	Electro-osmotic drag coefficient		
p	Total gas pressure	kPa	
$p_{W}$	Water vapor pressure	kPa	
R	Universal gas constant	J mol^−1^ K^−1^	8.314
$r_{local}$	Local mass transfer resistance per unit surface area of pt	10^−3^ s m^−1^	
S	Quality source term	kg m^−3^ s^−1^	
$s_{lq}$	Liquid water saturation		
T	Absolute temperature	K	0
t	Time	s	
wt%	Pt mass fraction in Pt/C mixture		0.6
x	The ratio of carbon surface normalized by Pt surface		1
$y_{bare}$	The volume fraction of bare carbon		0
$\alpha_{a/c}$	Anode/Cathode charge transfer coefficient		0.5/0.8
δ	Equivalent thickness of ionomer film	10^−8^ m	
ε	Volume fraction		
$\eta_{act}^{c}$	Cathodic overpotential	V	
θ	contact angle	°	105
$\theta_{PtOH}$	Pt oxide-coverage		
λ	Water content		
μ	Velocity of flow	L min^−1^	
μ	Gas flow rate	L min^−1^	
$\rho_{\;}$	Density	kg m^−3^	
σ	Surface tension of liquid water	N m^−1^	0.0644
$\phi_{\;}$	Potential	V	
ω	Volume fraction of ionomers		0.25
a	Anode		
act	Activation		
CL	Catalyst layer		
c	Cathode		
eff	Effective		
eq	Equilibrium		
GDL	Gas diffusion layer		
g	Gas phase		
in	Inlet		
ion	Ionic		
l	Liquid phase		
s	Solid phase		
W	Water		
