# Supplementary material for: Experimental and Numerical Simulation Study of Oxygen Transport in Proton Exchange Membrane Fuel Cells at Intermediate Temperatures (80 °C–120 °C)
Source: Membranes (Basel). 2024 Mar 22;14(4):72. doi: 10.3390/membranes14040072 (PMC11052493; doi:10.3390/membranes14040072)
Supplement: Supplementary file 1 [file membranes-14-00072-s001.zip › membranes-2874296-supplementary.pdf]

Experimental and numerical simulation study of oxygen transport in proton exchange membrane fuel cells at intermediate temperatures (80°C-120°C)

Jian Zhang[1,2], Yunfei Zhang[1], Zhengrui Xiao[1], Jinting Tan[1,2], Haining Zhang[1,2,], Jun Yu[1,2,*]

[1]State Key Laboratory of Advanced Technology for Materials Synthesis and Processing, Wuhan University of Technology, Wuhan 430070, China

[2]Key Laboratory of Fuel Cell Technology of Hubei Province, Wuhan University of Technology, Wuhan 430070, China

*Correspondence: yujun@whut.edu.cn

Polarization curve tests

Polarization curve tests were performed on a HEPHAS Mini-125s fuel cell test bench. The anode was supplied with H2 and the cathode with different percentages of O_2 (0.5%, 1%, 1.5%, 2%, 4%, 8%, 12%, 16%, 21%, diluted with N_2). Both the cathode and anode were supplied with a constant flow rate of hydrogen at 1600 mL/min and a total flow rate of diluted oxygen at 4000 mL/min. The use of high flow rates at both the anode and cathode is intended to ensure that the reaction consumption is small compared to the gas flowing into the cell, thus minimizing the effect of changes in gas composition on the experiment. The operating conditions of the cell were set at 80°C, 100°C, and 120°C, with back pressures of 100 kpa, 150 kpa, and 200 kpa. The humidity of the energized gas was controlled at 43%. Since we were using high gas flow rates and small oxygen percentages, even assuming that all water was present at the cathode, the cathode gas RH changed by only a few percent (the water production rate and the electroosmotic transfer rate were nearly two orders of magnitude lower than the inlet air rate), so we can assume that the humidity of the PEMFC remains constant throughout the experiment.

Electrochemical measurements

The cyclic voltammetry (CV) curve was obtained at a temperature of 30°C with a relative humidity of 100%. Hydrogen gas was provided to both the anode, serving as the reference electrode and counter electrode, while nitrogen gas was supplied to the cathode, functioning as the working electrode. The gas flow rates for both the cathode and anode were fixed at 200 ml/min. The voltage scanning range was 0.075 V to 1.2 V, with a scanning rate of 20 mV/s. Prior to testing, a 30-minute ventilation period was implemented to eliminate residual air. In the electrochemical impedance spectroscopy (EIS) test, the anode was supplied with hydrogen, the cathode was supplied with 21% oxygen, the constant voltage was maintained at 0.65 V, the frequency ranged from 10,000 Hz to 0.01 Hz, and the alternating current (AC) disturbance current was controlled at 10% of the direct current (DC) current.

Derivation of local oxygen transport resistance

The interfacial transmission resistance, diffusion resistance of ionomer film and the interfacial transmission resistance of platinum can be expressed as

$$R_{\text{ion,int}} = k_1 \frac{\delta_{\text{ion}}}{D_{O_2,\text{ion}}}. \quad \text{(S1)}$$

$$R_{\text{ion}} = \frac{\delta_{\text{ion}}}{D_{O_2,\text{ion}}} \quad \text{(S2)}$$

$$R_{\text{Pt,int}} = k_2 \frac{\delta_{\text{ion}}}{D_{O_2,\text{ion}}} \quad \text{(S3)}$$

Among them, k_1 and k_2 are the interface resistance coefficients of the ionomer film surface and the platinum particle surface respectively, and their values are 8.5 and 25 respectively[1], $D_{O_2,\text{ion}}$ is the diffusion rate of oxygen in the ion membrane, δ_{ion} is the thickness of the ionomer film can be expressed by the following formula

$$\delta_{\text{ion}} = ((\frac{\varepsilon_{\text{ion}}}{\varepsilon_{\text{C}}} + 1)^{1/3} - 1) r_{\text{C}} \quad \text{(S4)}$$

Among them, ε_{ion} is the volume fraction of ionomer, ε_{C} is the volume fraction of carbon particles, r_{C} is the radius of carbon particles. Their respective calculation formulas are as follows:

$$\varepsilon_{\text{C}} = \frac{1 - \varepsilon_{\text{CCL}} - \varepsilon_{\text{Pt}}}{1 + \rho_C (I/C)/\rho_{\text{ion}}} \quad \text{(S5)}$$

$$\varepsilon_{\text{ion}} = 1 - \varepsilon_{\text{CCL}} - \varepsilon_{\text{Pt}} - \varepsilon_{\text{C}} \quad \text{(S6)}$$

Among them, ε_{CCL} is the porosity of CCL, ε_{Pt} is the volume fraction of Pt particles, ρ_{C} is the density of carbon particles, and ρ_{ion} is the density of ionomer. The calculation formula for ε_{Pt} is:

$$\varepsilon_{\text{Pt}} = \frac{m_{\text{Pt}}}{\delta_{\text{CCL}} \rho_{\text{Pt}}} \quad \text{(S7)}$$

Where m_{Pt} is the Pt loading of CCL, and δ_{CCL} is the thickness of CCL.

The diffusion rate of oxygen in the ionomer film $D_{O_2,\text{ion}}$ can be calculated by the following formula:

$$D_{O_2,\text{ion}} = 1 \times 10^{-10} [0.1543(\text{T} - 273) - 1.65] \quad \text{(S8)}$$

Considering the diffusion path of oxygen in the ionomer, the effective length δ_{eff} is introduced and expressed as[1]:

$$\delta_{\text{eff}} = \frac{A_{\text{ion}}^{\text{eff}}}{A_{\text{Pt}}^{\text{eff}}}\delta_{\text{ion}} \tag{S9}$$

Where $A_{\text{ion}}^{\text{eff}}$ and $A_{\text{Pt}}^{\text{eff}}$ represent the effective ionomer film surface area and the effective platinum surface area respectively, and their calculation formulas are as follows:

$$A_{ion}^{\text{eff}} = \frac{4\pi(r_{\text{C}} + \delta_{\text{ion}})^2}{n_{\text{Pt}}} \tag{S10}$$

$$A_{Pt}^{\text{eff}} = 4\pi {r_{\text{Pt}}}^2(1 - \theta_{\text{PtOH}}) \tag{S11}$$

Among them, n_{Pt} is the number of platinum particles in Pt/C, r_{Pt} is the radius of the Pt particles, and θ_{PtOH} is the Pt oxide coverage, which are respectively defined as

$$\theta_{\text{PtOH}} = \frac{\exp\left(\frac{\alpha_a F\left(\eta_{\text{act}}^c + E_r - \phi_{eq}\right)}{RT}\right)}{\exp\left(\frac{\alpha_a F\left(\eta_{\text{act}}^c + E_r - \phi_{eq}\right)}{RT}\right) + \exp\left(-\frac{\alpha_c F\left(\eta_{\text{act}}^c + E_r - \phi_{eq}\right)}{RT}\right)} \tag{S12}$$

$$n_{\text{Pt}} = \frac{\rho_{\text{C}}}{\rho_{\text{Pt}}}(\frac{r_{\text{C}}}{r_{\text{Pt}}})^3(\frac{wt\%}{1 - wt\%}) \tag{S13}$$

In formula (20), α_a is the anode transfer coefficient formed by Pt oxide, α_c is the cathode transfer coefficient formed by Pt oxide, F is Faraday's constant, R is the ideal gas constant, T is the temperature in Kelvin, η_{act}^c corresponds to the cathode overpotential, E_r is the thermodynamic reversible potential, ϕ_{eq} is the equilibrium potential, which is fixed at 0.76V in this study. The calculation formulas for the cathode overpotential $\eta_{\text{act}}^{\text{c}}$ and the thermodynamic reversible potential E_r are respectively:

$$\eta_{\text{act}}^{\text{c}} = \phi_{\text{ele}} - \phi_{\text{ion}} - E_{\text{r}} \tag{S14}$$

$$E_r = 1.229 - 0.9 \times 10^{-3}(T_0 - 298) + \frac{RT_0}{2F}(\ln p_{\mathrm{H_2}}^{\mathrm{in}} + \frac{1}{2} p_{\mathrm{O_2}}^{\mathrm{in}}) \quad \text{(S15)}$$

where ϕ_{ele} is the potential at the end of the cathode BP surface, that is, the output voltage, ϕ_{ion} is the potential of the ionomer film, $p_{\mathrm{H_2}}^{\mathrm{in}}$ is the inlet hydrogen pressure, $p_{\mathrm{O_2}}^{\mathrm{in}}$ is the inlet oxygen pressure, T_0 is the operating temperature.

In formula (21), ρ_{Pt} is the density of Pt particles, $wt\%$ denotes the weight percentage of Pt in the Pt/C catalyst, with the calculation formula being:

$$wt\% = \frac{\rho_{\mathrm{Pt}}\varepsilon_{\mathrm{Pt}}}{\rho_{\mathrm{Pt}}\varepsilon_{\mathrm{Pt}} + (1 - y_{\mathrm{bare}})\rho_{\mathrm{C}}\varepsilon_{\mathrm{C}}} \quad \text{(S16)}$$

Among them, y_{bare} is the mass fraction of bare carbon with zero loading of Pt. In this experiment, the y_{bare} value is fixed at 0.

Substituting formula (18) to formula (24) into formula (17), we can get:

$$\delta_{\mathrm{eff}} = \frac{(r_{\mathrm{C}} + \delta_{\mathrm{ion}})^2}{r_{\mathrm{Pt}}^2(1 - \theta_{\mathrm{PtOH}})} \frac{\rho_{\mathrm{Pt}}}{\rho_{\mathrm{C}}} (\frac{r_{\mathrm{Pt}}}{r_{\mathrm{C}}})^3 (\frac{1 - wt\%}{wt\%}) \delta_{\mathrm{ion}} \quad \text{(S17)}$$

Hence, when considering the influence of the transmission path on the diffusion resistance of the ionomer film, the diffusion resistance of the ionomer and the interfacial transmission resistance of platinum can be modified as[2]

$$R_{\mathrm{ion}}^{\mathrm{eff}} = \frac{\delta_{\mathrm{ion}}}{D_{\mathrm{O_z,ion}}} \frac{x\rho_{\mathrm{Pt}} r_{\mathrm{Pt}} (r_{\mathrm{C}} + \delta_{\mathrm{ion}})^2 (1 - wt\%)}{\rho_{\mathrm{C}} r_{\mathrm{C}}^3 wt\% (1 - \theta_{\mathrm{PtOH}})} \quad \text{(S18)}$$

$$R_{\mathrm{Pt,int}}^{\mathrm{eff}} = k_2 \frac{\delta_{\mathrm{ion}}}{D_{\mathrm{O_z,ion}}} \frac{x\rho_{\mathrm{Pt}} r_{\mathrm{Pt}} (r_{\mathrm{C}} + \delta_{\mathrm{ion}})^2 (1 - wt\%)}{\rho_{\mathrm{C}} r_{\mathrm{C}}^3 \mathrm{wt}\% (1 - \theta_{\mathrm{PtoH}})} \quad \text{(S19)}$$

where x is the fraction of total catalyst particles, and the calculation formula is:

$$x = \frac{(1 - wt\%)(1 - y_{\mathrm{bare}})}{1 - wt\%(1 - y_{\mathrm{bare}})} \quad \text{(S20)}$$

Model geometric parameters

The geometric parameters of the fuel cell structure used in the modeling process are shown in Table S1.

Table S1 Geometrical parameters of the fuel cell structure with triple serpentine flow channels

Cell Structure Characteristic Parameters			
Parameters	Value	Parameters	Value
Cell length (mm)	50	Cell width (mm)	50
Bipolar plate height (mm)	2	Bipolar plate width (mm)	0.83
Channel width (mm)	0.83	Channel height (mm)	1
GDL thicknesses (μm)	110	MPL thicknesses (μm)	3
Cathode CL thicknesses (μm)	10	Anode CL thicknesses (μm)	10

Cell electrochemical parameters			
Parameters	Value	Parameters	Value
Anode reference exchange current density $j_{0,ref}^{a} (A/m^2)$	100000	Cathode reference exchange current density $j_{0,ref}^{c} (A/m^2)$	100
Anode transfer coefficient	$\alpha_a^{an} = 0.7$ $\alpha_c^{an} = 0.3$	Cathode transfer coefficient	$\alpha_a^{cat} = 0.35$ $\alpha_c^{cat} = 0.65$
Anode concentration factor γ_a	1	Cathode concentration factor γ_c	1
Hydrogen reference concentration $(kmol/m^3)$	1	Oxygen reference concentration $(kmol/m^3)$	1
Anode entropy change $\Delta S_a (J/(kmol \cdot K))$	0	Cathode entropy change $\Delta S_c (J/(kmol \cdot K))$	-163300
Reversible electromotive force $E_{rev}(V)$	1.15	Active area (m^2)	0.0025

Gaseous Diffusion Layer			
Parameters	Value	Parameters	Value
Porosity	0.78	Intrinsic penetration (m^2)	8×10^{-12}
Contact angle (°)	130	Densities (kg/m^3)	440
Specific heat capacity $(J/(kg \cdot K))$	710	Heat conductivity $(W/(m \cdot K))$	1.7
Conductivity (S/m)	5000		

Catalytic Layer

Parameters	Value	Parameters	Value
Porosity	0.78	Intrinsic penetration (m^2)	3×10^{-14}
Contact angle (°)	120	Densities (kg/m^3)	1000
Specific heat capacity $(J/(kg \cdot K))$	3300	Heat conductivity $(W/(m \cdot K))$	0.27
Conductivity (S/m)	1000		

Microporous Layer			
Parameters	Value	Parameters	Value
Porosity	0.6	Intrinsic penetration (m^2)	5×10^{-13}
Contact angle (°)	140	Densities (kg/m^3)	440
Specific heat capacity $(J/(kg \cdot K))$	710	Heat conductivity $(W/(m \cdot K))$	1
Conductivity (S/m)	5000		

Bipolar Plates			
Parameters	Value	Parameters	Value
Conductivity (S/m)	5000	conductivity $(W/(m \cdot K))$	440
Specific heat capacity $(J/(kg \cdot K))$	710	Heat conductivity $(W/(m \cdot K))$	1

Proton Exchange Membranes			
Parameters	Value	Parameters	Value
Equivalent mass (kg/mol)	1	Intrinsic penetration (m^2)	1×10^{-13}
Conductivity (S/m)	1×10^{-16}	Densities (kg/m^3)	1980
Specific heat	2000	Heat	2

capacity $(J/(kg \cdot K))$	conductivity $(W/(m \cdot K))$

Boundary conditions and solution methods

The CFD software Ansys Fluent is utilized to solve the governing equations of section 3.2.2 in a double-precision format based on the finite volume method. Where the velocity-pressure coupling in the momentum equation is handled computationally using the SIMPLE algorithm, the specific interpolation function is the second-order windward method, and to ensure that the computation is stable enough to reach convergence, appropriate relaxation factors are applied to each control variable. The inlet/outlet and wall surfaces of the cell are set: the cell inlet boundary is set as mass flow control inlet, the cell outlet boundary is set as pressure control outlet, and the other wall surfaces are set as constant temperature wall surfaces. Adopt constant current control mode for calculation, set the anode to 0 potential and the cathode to working current density.

Model meshing

For simulation modeling, meshing is one of the most critical steps. Good or bad meshing directly affects the accuracy and speed of the calculation. Reasonable meshing can quickly produce simulation results under the premise of ensuring the accuracy of the results. Since different regions of the fuel cell have different structures and roles, the requirements for the grid will also be different, so it is necessary to perform a reasonable grid division for different regions according to the required resolution. This model divides the anode, cathode GDB, MPL, CL, and proton exchange membrane all into 284050 grids, totaling 3129550 rectangular grids.

Model Validation

This simulation of the proton exchange membrane fuel cell is calculated using the fluid simulation software Ansys Fluent, the polarization curves calculated by the created three-snake flow field model are compared with the polarization curves measured by the actual use of the three-snake flow field, and the results are shown in

Fig. S1.

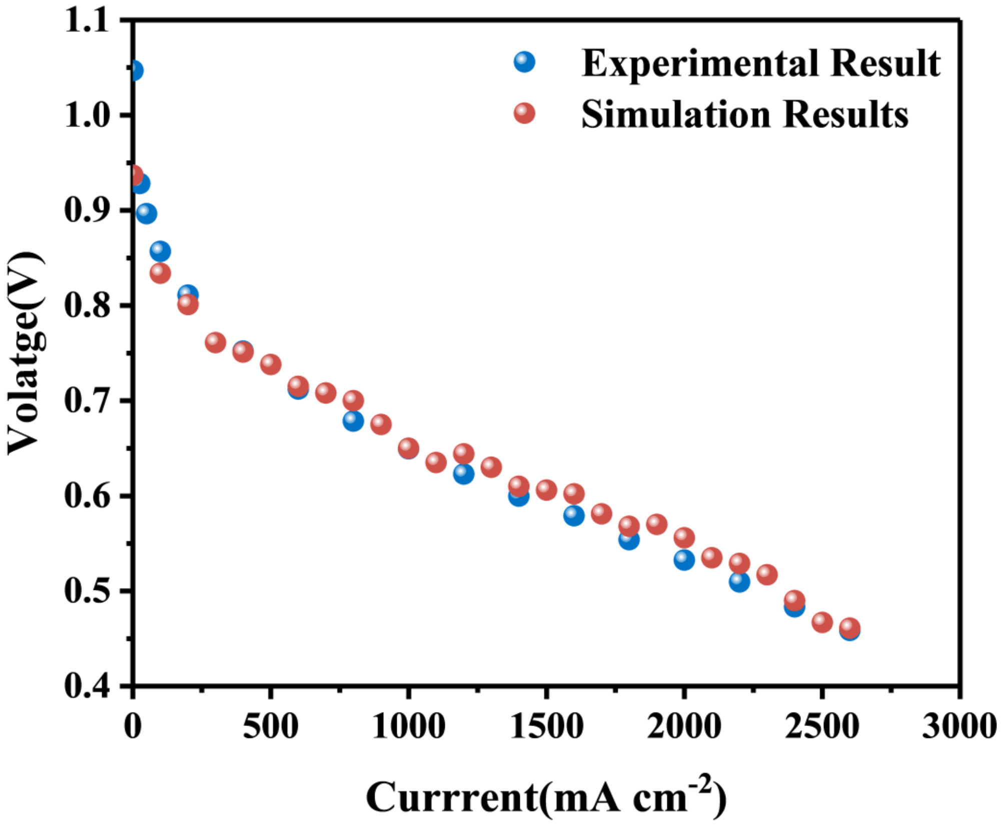

Fig. S1 Comparison of simulated and experimental data

When we validated the simulation, the conditions set for the numerical simulation were the same as the experimental ones (100°C, 150kpa, RH43%). From the figure we can see that the experimental data fit well with the simulation data, which verifies the accuracy of the simulation data and the established mathematical model is reliable.

Temperature cycle experiment

During the heating process from 80°C to 120°C, we measured polarization curves, Electrochemical Impedance Spectroscopy (EIS), and Linear Sweep Voltammetry (LSV) curves at 80°C, 100°C, and 120°C, respectively. After stabilizing the operation at 120°C for a period, we gradually cooled the battery to 80°C, during which we again measured the polarization curves, EIS, and LSV curves at 100°C and 80°C. The specific results are shown in Figure S2, where LSV measurements were conducted to evaluate the hydrogen permeation current density and assess the membrane's permeability to hydrogen gas, thereby examining any damage to the membrane after operation at 120°C.

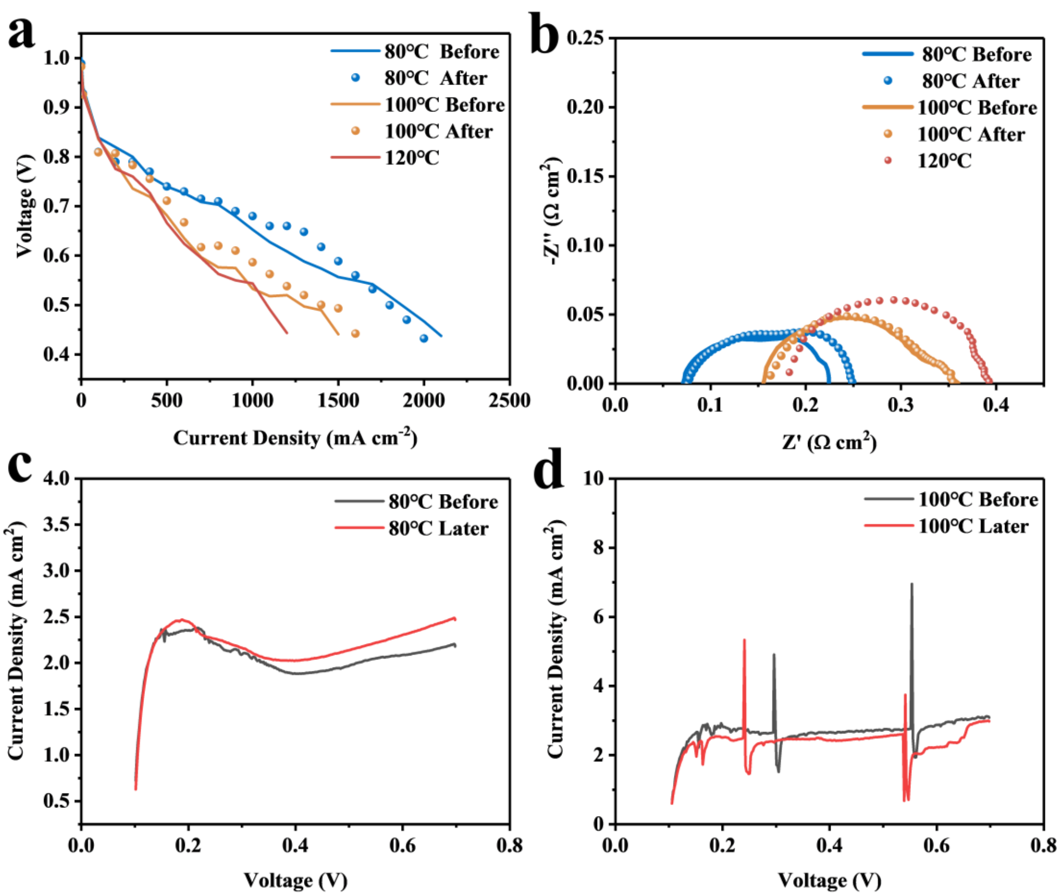

Figure S2. (a) Polarization curves of the cell at 80°C, 100°C and 120°C before and after operation at 120°C;(b) EIS at 80°C, 100°C and 120°C before and after operation of the cell at °C; (c) Experienced LSV before and after 120°C, at 80°C; (d) Experienced LSV before and after 120°C, at 100°C

From Figure S2(a), it can be observed that, after operating under conditions of 120°C, the open-circuit voltage (OCV) remained almost unchanged with a minimal decrease of only 0.01V, both at 80°C and 100°C. Furthermore, the overall trend of the polarization curves showed no significant difference. Analysis of the Electrochemical Impedance Spectroscopy (EIS) in Figure S2(b) indicated that the EIS spectra before and after the 120°C test overlapped almost completely, suggesting that the membrane's electrochemical performance remained stable after exposure to 120°C. This study was conducted according to the Chinese National Standard GB/T 20042.5-2009 for testing membrane electrode assemblies in proton exchange membrane fuel cells, measuring the hydrogen permeation current density. The results presented in Figures S2 (c) and S2 (d)

showed that the hydrogen permeation current density at different temperatures remained consistent before and after operation at 120°C, with no observed change in magnitude. This outcome indicates that the membrane material, even after operating at 120°C for a period, maintained its original structural and performance characteristics without significant damage.

The preliminary results of these experiments indicate that the Nafion membrane's data remained essentially unchanged after heating from 80°C to 120°C, stabilizing at 120°C for a period, and then cooling back to 80°C, demonstrating that the data obtained under current test conditions are stable and reliable. These data support our research on oxygen transport at intermediate temperatures.

References

1. Hao, L.; Moriyama, K.; Gu, W.; Wang, C.-Y. Modeling and Experimental Validation of Pt Loading and Electrode Composition Effects in PEM Fuel Cells. *J. Electrochem. Soc.* **2015**, *162*, F854, doi:10.1149/2.0221508jes.
2. Yoon, W.; Weber, A.Z. Modeling Low-Platinum-Loading Effects in Fuel-Cell Catalyst Layers. *J. Electrochem. Soc.* **2011**, *158*, B1007, doi:10.1149/1.3597644.
